# Publicly-funded biobanks and networks in East Asia

**DOI:** 10.1186/s40064-016-2723-2

**Published:** 2016-07-15

**Authors:** Sunhee Lee, Paul Eunil Jung, Yeonhee Lee

**Affiliations:** Korea National Research Resource Center (KNRRC), #324, Golden 50 Commemoration Hall, Seoul Women’s University, 621 Hwarang-ro, Nowon-gu, Seoul, 01797 Korea; Department of Horticulture, Biotechnology and Landscape Architecture, Seoul Women’s University, 621 Hwarang-ro, Nowon-gu, Seoul, 01797 Korea

## Abstract

With the enactment of the Nagoya Protocol, international competitions to secure biological resources are intensifying. Biobanking is one of the many attempts to preserve biological resources and their information for the use in future research and development. Asian countries, especially China, Japan, and Korea are very active in biobanking activities under the strategic plans coordinated by their governments. They also proactively established networks for biobanks of Asia to facilitate resource and expertise sharing. Biobanks of these countries should furthermore standardize operating procedures and diversify funding sources for establishing stable operation systems.

## Background

Since the adoption of the Convention on Biological Diversity (CBD) in 1992, biological resources have been increasingly considered as exclusive possessions of their originating countries rather than common assets of mankind. International competitions to secure bioresources have recently been overheated with the enactment of the Nagoya Protocol on access to genetic resources and benefit sharing (ABS) in 2014. According to the CBD provision, biological resources are genetic resources and organisms in parts or their entireties with actual or potential uses and values for mankind. They are more than simple collections of organisms—they are the source of academic and industrial advancement and exploitation.

Biobanking is one of the many attempts to conserve ecologically and scientifically valuable biological resources and their associated information for the advancement of life sciences. Biobanks both accept and provide viable (often culturable and replicable) organisms or their parts and information on their molecular and physiological characteristics. Since its first appearance in a scientific literature in 1990s (Hewitt and Watson 2013), the term “biobank” was often used narrowly to describe a repository of human specimens. As more scientists, policymakers, and other stakeholders of biobanks expanded the range of its definitions, the word now also accommodates non-human organisms, such as plants, animals, fungi, and bacteria. In recent online surveys, biobanking stakeholders concurred in including different organisms in the biobanking realm and further agreed that biobanks should follow standard operating procedures and distribute materials for scientific use (Hewitt and Watson 2013). Broader definitions of the term are also reflected in international guidelines and numerous national regulations, such as the guidelines of Organisation for Economic Co-operation and Development (OECD) and the laws of several European countries (Hewitt and Watson 2013).

Biobanking activities were recorded in Europe as early as 1890s (Day and Stacey 2008). Since then, numerous institutes were established in Europe and led the effort of conserving and distributing biological resources. For culture collections, European institutes outnumber the institutes of other continents by far in terms of volume and diversity of their collections. However, Japan, Korea, and China have recently seen a rapid increase in the new deposits. In terms of the numbers of new deposits, yearly aggregated amount of the three countries are comparable to the European aggregate (Smith et al. 2013).

In this paper, we observe the status of biobanks in Asian countries, with emphases on three East Asian countries, China, Japan, and Korea. Despite the recent rise of Asian countries in the biobanking field, lack of coordination may have precluded international scholars to identify and have access to Asian biobanks. Through this paper, we identify public biobanks of Asian countries and explain how they operate. Furthermore, we present major networks of Asia–Pacific biobanks and their functions.

## Biobanks by countries

### Korea

Prior to 1990, academic and clinical institutes in Korea primarily led the preservation efforts of biological resources. As worldwide interest in biological diversity and conservation heightened, the Korean government started to invest heavily in securing and utilizing biological resources for academic and industrial uses. In 1995, the Ministry of Science and Technology^1^ initiated a federal project to support and foster 5 research resource centers (RRCs; Lee and Lee 2009). This project has expanded to encompass 31 RRCs and 4 core centers, collectively managed by a central organization, Korea National Research Resource Center (KNRRC).

Korean public biobanks are operated by collaborative efforts of six government ministries (National Science and Technology Council Steering Committee 2015). Ministries collectively establish yearly plans for biological resource management, in order to prioritize policies for biological conservation, reduce risk factors in biological diversity, uphold sustainable use of ecosystem services, and strengthen international collaboration on conservation and research efforts. Under these joint objectives, each ministry has established responsible agencies to manage and support subordinate biobanks in accordance with their ministerial focus and interest.

Human biobanks are administered by the three ministries, the Ministry of Health and Welfare (MOHW), the Ministry of Food and Drug Safety (MFDS), and the Ministry of Science, ICT, and Future Planning (MSIP). Korea Centers for Disease Control and Prevention (KCDC), under the administration of MOHW, launched the Korea Biobank Project (KBP) in 2008. Through this project, KCDC has planned to collect specimens from 200,000 patients and 300,000 general population (1st term, from 2008 to 2012) and to establish Korean Biobank Network for specimen distribution (2nd term, from 2013 to 2015; Cho et al. 2012). Until now, National Biobank of Korea (NBK), along with 17 regional biobanks of university hospitals and 2 network-affiliated hospitals, has accumulated human serum, plasma, DNA, and other specimens from 328,000 patients (as of December 2015). NBK also accommodates population-based specimens collected from 384,000 people as part of regional cohort studies (Centers for Disease Control & Prevention, Korea 2016).

MSIP administers exceptionally diverse biobanks, unlike other ministries, in addition to the human biobanks. In accordance with the ministerial goal to foster scientific research (Ministry of Science, ICT and Future Planning 2015), three institutes, the Korea Research Institute of Bioscience and Biotechnology (KRIBB), the National Science Museum, and the Korea National Research Resource Center (KNRRC), endeavor to secure diverse research resources, including biological and non-biological resources. KRIBB manages 1 human biobank and 5 biobanks of other organisms, including animals, plants, and microbes (Korea Research Institute of Bioscience and Biotechnology 2014). The institute has set up the International Biological Material Research Center (IBMRC) for international research collaboration and collection of novel specimens. The National Science Museum houses 700,000 specimens of 9100 terrestrial and marine species (Korean Bio-resource Information System 2015). KNRRC accommodates 31 diverse biobanks of biological and non-biological research resources. The center has established best practice guidelines and integrated resource inventory system for biobanks to ensure the quality of resources and their information. Additionally, KNRRC hosts seminars and forums to educate biobank directors, staffs, and other biobanking stakeholders.

Other federal ministries handle more specialized biobanks, consistent with their ministerial responsibilities. Under the Ministry of Agriculture, Food, and Rural Affairs (MAFRA), the National Institute of Agricultural Sciences (NAS) and the National Institute of Animal Science (NIAS) support crops and livestock research and preservation implemented by laboratories of universities, local governments, and government-funded organizations. Korea Forest Service, another important agency of MAFRA, manages plant, insect, and microbial resources via National Institute of Forest Science (NIFoS), Korea Forest Seed and Variety Center (NFSV), and Korea National Arboretum (National Science and Technology Council Steering Committee 2015). Ministry of Oceans and Fisheries (MOF) appointed 14 laboratories of universities and research institutes as marine bioresource banks. Marine Biodiversity Institute of Korea was established in 2014 for public exhibition and preservation of marine organisms. The institute has collected more than 400,000 specimens which are as diverse as bacteria, fungi, algae, and vertebrates. Ministry of Environment (ME) operates the National Institute of Biological Resources (NIBR) for wildlife conservation and protection of endangered species.

Federal ministries will continue the collaborative works for management of biological resources until 2020 as specified in the master plan laid out by the ministries in 2011. Table 1 lists publicly funded biobanks in Korea.

**Table 1:** Biobanks in Korea

Name of institute	Website	Resources
Korea National Research Resource Center (KNRRC)	www.knrrc.or.kr/english	Animal, plant, human-origin, microorganism
Korea Research Institute of bioscience & Biotechnology	www.kribb.re.kr/eng/sub02/sub02_05.jsp	Animal, plant, human-origin, microorganism
Marine Biodiversity Institute of Korea	www.mabik.re.kr/html/en/	Animal, plant, microorganism
National Institute of Biological Resources	www.nibr.go.kr/eng	Plant, microorganism, vertebrata, insect specimen
National Science Museum	www.science.go.kr/english/index.html	Animal, plant, DNA
RDA-Genebank Information Center	www.genebank.go.kr/eng/	Plant germplasm, DNA, microorganism, sequence, silkworm, insect
*Animal*		
Aging Tissue Bank	atb.knrrc.or.kr	Fresh frozen tissue, serum, animal model
Animal Bio Resources Bank	www.abrb.or.kr	Animal secretion, physiologically active substance
Arthropods of Medical Importance Resource Bank	amib.knrrc.or.kr	Animal extract, fresh frozen tissue, specimen, DNA
Bovine Genome Resource Bank	bgrb.knrrc.or.kr	Paraffin block, serum, whole blood, DNA
Korean Coral Resource Bank	www.coralbank.co.kr	cDNA library, DNA
Laboratory Animal Resource Center	mouse.kribb.re.kr	Mouse, fertilized egg
Marine Arthropod Depository Bank of Korea	www.madbk.org	Specimen
Marine Echinoderm Resources Bank of Korea	merbk.syu.ac.kr/main.do	Specimen, sequence, DNA
Marine Fish Resource Bank of Korea	cms.pknu.ac.kr/mfrbk	Specimen, DNA
Marine Mollusk Resource Bank of Korea	www.mmrbk.org	Specimen, clone library, DNA
National Primate Research Center	eng.primate.re.kr	Nucleic acid, tissue, cell, brain tissue
Neurogenic Laboratory & Neuromarker Resource Bank	www.brainprism.org	Specimen, protein, nucleic acids, BAC
Parasite Resource Bank	www.parasite-bank.or.kr	Tissue, egg, specimen, DNA, serum
Zebrafish Organogenesis Mutant Bank	zomb.knrrc.or.kr	Live animal, nucleic acids, sequence
*Microorganism*		
Bacteriophage Bank	www.phagebank.or.kr	Phage
Center for Fungal Genetic Resources	genebank.snu.ac.kr	Fungi, DNA
Culture Collection of Antimicrobial Resistant Microbes	www.ccarm.or.kr	Bacteria, fungi
Culture Collection of Mushrooms	ccm.knrrc.or.kr	Fungal culture
Extract Collection of Useful Microorganism	www.ecum.or.kr	Extract
*Helicobacter pylori* Korean Type Culture Collection	hpktcc.knrrc.or.kr	Bacteria, clone, cell strains
Korea Bank for Pathogenic Viruses	kbpv.knrrc.or.kr	Serum, virus
Korea Environmental Microorganisms Bank	www.kbem.or.kr	Bacteria
Korea Mushroom Resource Bank	kmrb.knrrc.or.kr	Fungal culture
Korea Veterinary Culture Collection	kvcc.kahis.go.kr	Fungi, bacteria, virus, parasite, clone, serum
Korean Collection for Oral Microbiology	kcom.knrrc.or.kr	Bacteria
Korean Collection for Type Cultures	kctc.kribb.re.kr/English/index.aspx	Bacteria, fungi, microalgae, DNA
Korean Lichen and Allied Bioresource center	kolabic.knrrc.or.kr	Fungi, microalgae, extract
Marine & Extreme Bioresources Collections	www.mebic.re.kr/mebic_l1/eng	Bacteria, algae, benthos
Marine Fungal Resource Bank	mfrb.snu.ac.kr	Fungi, DNA
Metagenome Resource Bank	mgrb.knrrc.or.kr	Sequence
Mushroom bank	www.genebank.go.kr/pm_m/main.jsp	Specimen
Myxobacteria Bank	myxobank.knrrc.or.kr	Myxobacteria
National Culture Collection for Pathogens	nccp.cdc.go.kr	Pathogenic microorganism, DNA, RNA, plasmid, clone
Plant Virus Genebank	www.virusbank.org	Virus, antibody, sequence
Smart Microbial Carbohydrate lab	smclab.konkuk.ac.kr	Microbial carbohydrate
Waterborne Virus Bank	www.wava.or.kr	Virus
*Plant*		
Brassica Resource Bank	brb.knrrc.or.kr	Seed
Center for the Korea Potato Genetic Resources	kpgr.knrrc.or.kr	Plant, seed
Ginseng bank	gb.knrrc.or.kr	Plant, plant extract, seed, cell line, DNA
International Biological Material Research Center	www.ibmrc.re.kr	Plant
Korea Bioactive Natural Material Bank	kbnmb.knrrc.or.kr	Plant, plant extract
Korea Plant Extract Bank	extract.kribb.re.kr	Plant, plant extract
Korea Seed & Variety Service	www.seed.go.kr/english	Seed
Korean Marine Plant Collection	www.kmpc.kr	Specimen, culture, DNA
Marine Brown Algae Resources Bank	mbrb.chosun.ac.kr/main.do	Specimen, extract, culture, DNA
Marine Green Algal Resources Bank	mgarb.pknu.ac.kr/main.do	Specimen, culture, DNA
Medicinal Plant Resources Bank	mprb.knrrc.or.kr	Plant, extract, DNA
National Forest Seed and Variety Center	www.kfsv.go.kr	Seed, clone, specimen
National Institute of Agricultural Sciences	www.naas.go.kr/english	Plant, seed
Plant DNA bank in Korea	pdbk.korea.ac.kr	DNA
*Human*-*origin*		
Human Serum Bank	hsb.knrrc.or.kr	Serum
Korea Prostate Bank	www.prostatebank.or.kr	Tissue, plasma, serum, urine, DNA
Korean Cell Line Bank	cellbank.snu.ac.kr/english	Cell line
Korean Gynecologic Cancer bank	kgcb.or.kr	Serum, body fluid, cell line, tissue, plasma, saliva, urine
Korean Human Gene Bank	genbank.kribb.re.kr	cDNA, gene set, library, clone
Korean Leukemia Cell & Gene Bank	www.klcgb.or.kr/eng	Cell line, DNA
Liver Cancer Specimen Bank	lcsb.knrrc.or.kr	Tissue, plasma
National Biobank of Korea	kbn.cdc.go.kr	Serum, plasma, urine, buffy coat, DNA
Seoul National University Hospital Biomedical Research Institute	en.bri.snuh.org	Serum, plasma, buffy coat, nucleic acid, B cell, urine, lymphocytes, blood, tissue
Wonkwang University Hospital	med.wku.ac.kr/?page_id=507	Tissue, blood, DNA

### Japan

Japan has extensive specimen collections of diverse organisms which often have been managed by small-sized laboratories of academic or governmental institutes. The Japanese government has recently reorganized the management and funding scheme for biobanks as the government heavily emphasizes quality management of biological resources.

In 2015, the Cabinet Office of Japan established Japan Agency for Medical Research and Development (AMED) as a “control tower” for medical research and development (R&D; Japan Agency for Medical Research and Development 2015). Previously, medical research in Japan was administered by three different ministries, Ministry of Education, Culture, Sports, Science and Technology (MEXT), Ministry of Health, Labour, and Welfare (MHLW), and Ministry of Economy, Trade and Industry (METI; Sano 2015). Each ministry had implemented different funding schemes which added burdens to medical researchers. As a part of the national revitalization strategy in 2013, the Cabinet acknowledged the importance of medical R&D and decided to create a “headquarters of healthcare policy,” modeled after National Institutes of Health (NIH) of the United States (Hishiyama 2015; Japan Agency for Medical Research and Development 2015). This initiative was developed into the creation of AMED. While three ministries remain as key stakeholders, AMED now works as an integrated funding agency between researchers and ministries. The government anticipates that fast-tracked funding and clinical trial process will help researchers to engage more in research and develop novel treatments for Japanese population.

The Japanese government is currently interested in personalized treatments enabled by advance in genomic research. For collection of human specimens, MEXT and MHLW collaborate in managing human biobank networks of BioBank Japan (BBJ), National Center Biobank Network (NCBN), and Tohoku Medical Megabank Organization (ToMMo) biobank project (Furuta 2014). BBJ is a disease-oriented biobank which embarked in 2003 (Okamura et al. 2014). From its first cohort study (2003–2007), DNA, serum, and medical records of 200,000 patients were collected. RIKEN Center for Integrative Medical Sciences (IMS) and the Institute of Medical Science at the University of Tokyo (IMSUT) have teamed up for genomic analyses of specimens and data management. These institutes are securing additional DNA specimens and medical records from 100,000 patients of the second cohort (2013–2018). NCBN is also a network for disease-oriented biobanks, in collaboration of six centers specialized in different diseases, including cancer, neurology, cardiovascular diseases. While each center individually operates its own hospital and research institutes, they perform joint research through the shared platform among the centers. ToMMo biobank is a population-based institute, unlike the previous two networks, and created for the long-term study of people who suffered the 2011 Tohoku earthquake. With the support of the latest IT technology, this biobank aims to collect medical and genomic data of 150,000 individuals from 10,000 affected households with three generations (Matsui and Tashiro 2014).

National BioResource Project (NBRP) is an initiative managed by AMED for integrated and standardized management of biological resources (Nagai et al. 2015). Under the supervision of MEXT, the program commenced in 2002 to upgrade biobanking systems and facilities scattered throughout Japan. The program is at its third phase (2012–2016) of 5-year term and recently faced management transfer to AMED along with the opening of the agency. NBRP currently supports 29 facilities handling specimens and living organisms for scientific research. The project also emphasizes genome-enabled research to enhance the values of collected resources and specimen databases to better serve researchers.

For agricultural conservation efforts, Genebank Project has been implemented by the National Institute of Agrobiological Sciences (NIAS), under the supervision of the Ministry of Agriculture, Forestry and Fisheries (MAFF; National Institute of Agrobiological Sciences 2014; NIAS Genebank 2015). NIAS has accumulated 215,000 plant, 28,000 microbe, and 1000 animal accessions through the project and established online databases to provide their data to researchers. The project also aims to strengthen international cooperation and secure foreign genetic resources through exchange programs, joint research, and symposiums.

National Institute of Technology and Evaluation’s Biological Resource Center (NBRC) of METI specializes in collection and preservation of microorganisms. NBRC provides services of domestic and international distributions and patent deposits and online databases for microbes with industrially useful properties (National Institute of Technology and Evaluation 2015b). The BRC also hosted the first meeting of Asian Consortium for the Conservation and Sustainable Use of Microbial Resources (ACM) and has strived to build cooperative relationships with other Asian countries (Asian Consortium For the Conservation and Sustainable Use of Microbial Resources 2015a; National Institute of Technology and Evaluation 2015a).

In summary, the Japanese government aims to strengthen life science research by the establishment of a dedicated funding agency and strategic support on biobanks handling useful resources for industrialization. Table 2 lists government-funded biobanks in Japan.

**Table 2:** Biobanks in Japan

Name of institute	Website	Resources
BioResource Center, RIKEN	en.brc.riken.jp	Animal, microorganism, plant, human-origin
DNA Bank, RIKEN BRC^a^	dna.brc.riken.jp	Human, mouse, *S. pombe* clones
Japan Genetic Resources	www.shigen.nig.ac.jp/wgr/jgr/jgrUrlList.jsp	Animal, microorganism, plant
National BioResource Project (NBRP)	www.nbrp.jp	Animal, microorganism, plant, human-origin
National Institute of Genetics	www.nig.ac.jp/nig/	Animal, microorganism, plant, human-origin
NIAS Genebank	www.gene.affrc.go.jp	Animal, microorganism, plant, DNA
*Animal*		
Avian Bioscience Research Center^a^	www.agr.nagoya-u.ac.jp/~nbrp/en/	Chicken and quail: blood, egg, DNA
Brain Science Institute, RIKEN^a^	shigen.nig.ac.jp/zebra/	Zebrafish
*C. elegans*, Department of Physiology, TWMU^a^	shigen.nig.ac.jp/c.elegans/	Nematode
ENU Mutants	ja.brc.riken.jp/lab/gsc/mouse	Mouse mutant
Experimental Animal Division, RIKEN BRC^a^	mus.brc.riken.jp/en/	Mouse
Fly stocks of National Institute of Genetics (NIG-FLY)	shigen.nig.ac.jp/fly/nigfly	*Drosophila*
Institute of Laboratory Animals^a^	www.anim.med.kyoto-u.ac.jp/nbr	Rat
JCRB Laboratory Animal Resource Bank	animal.nibiohn.go.jp	Mouse
KYORIN-Fly	shigen.nig.ac.jp/fly/kyorin/	*Drosophila*
Kyoto Stock Center DGGR^a^	www.dgrc.kit.ac.jp	*Drosophila*
Medaka BioResource Unit, National Institute for Basic Biology^a^	shigen.nig.ac.jp/medaka	Medaka
Misaki Marine Biological Station	marinebio.nbrp.jp/oxycomanthus	*Oxycomanthus japonicus*
Mouse Genetic Resources	shigen.nig.ac.jp/mouse/nig	Mouse
National Institute for Physiological Sciences^a^	nihonzaru.jp	Japanese macaque
National Research Institute of Aquaculture	nria.fra.affrc.go.jp	Live animal, pathogen
*Paramecium*, Faculty of Science, Yamaguchi University^a^	nbrpcms.nig.ac.jp/paramecium	*Paramecium*
Shimoda Marine Research Center^a^	marinebio.nbrp.jp/ciona	*Ciona intestinalis*
Silkworm, Faculty of Agriculture, Kyushu University^a^	silkworm.nbrp.jp	Silkworm
Institute for Amphibian Biology^a^	home.hiroshima-u.ac.jp/amphibia/xenobiores	*Xenopus* (tropical clawed frog)
*Microorganism*		
*Bacillus subtillis*, National Institute of Genetics^a^	shigen.nig.ac.jp/bsub	Bacteria, clone
Cellular slime molds, Faculty of Life and Environmental Sciences, University of Tsukuba^a^	nenkin.nbrp.jp	Slime mold, clone, DNA
*E. coli* strain, National Institute of Genetics^a^	shigen.nig.ac.jp/ecoli/strain	Clone, plasmid, phage
Japan Collection of Microorganisms, RIKEN BRC^a^	jcm.brc.riken.jp	Microorganism
JapoNet, National Institute of Genetics	night.nig.ac.jp/labs/MicroGen/japonet	*Schizosaccharomyces japonicus* yeast, plasmid
Microbial Culture Collection, National Institute for Environmental Studies	mcc.nies.go.jp	Microorganism
NITE Biological Resource Center	www.nite.go.jp/en/nbrc	Microorganism
Research Center for Pathogenic Fungi and Microbial Toxicoses^a^	pathogenic.lab.nig.ac.jp	Pathogenic fungi, bacteria, protozoa
Yeast Genetic Resource Center^a^	yeast.lab.nig.ac.jp	Yeast, clone, plasmid, DNA
*Plant*		
Algae, National Institute for Environmental Studies^a^	shigen.nig.ac.jp/algae	Algae culture, DNA
Barley and Wild Plant Resource Center^a^	shigen.nig.ac.jp/barley	Seed, DNA
Chrysanthemum, Laboratory of Plant Chromosome and Gene Stock, Hiroshima University^a^	shigen.nig.ac.jp/chrysanthemum	Seed
Experimental Plant Division, RIKEN BRC^a^	epd.brc.riken.jp/en/	*Arabiodopsis*: cultured cell, seed, gene
Frontier Science Research Center^a^	www.legumebase.brc.miyazaki-u.ac.jp	*Lotus japonicus*, *Glycine max*/*soja*: seed, vector, DNA; bacteria
Komugi, Laboratory of Genetics, Kyoto University^a^	shigen.nig.ac.jp/wheat/komugi	Wheat: seed, microarray, DNA
Morning Glory, Faculty of Science, Kyushu University^a^	shigen.nig.ac.jp/asagao	Seed, DNA
Oryzabase, National Institute of Genetics^a^	shigen.nig.ac.jp/rice/oryzabase	Rice: wild and mutant strain
Tohoku Univ. *Brassica* Seed Bank	www.agri.tohoku.ac.jp/pbreed/Seed_Stock_DB/Stock_English_top.html	Seed
Tomato, Gene Research Center^a^	tomato.nbrp.jp	Plant, DNA
*Human*-*origin*		
BioBank Japan	biobankjp.org	Serum, DNA
Cell Engineering Division – Cell Bank, RIKEN BRC^a^	cell.brc.riken.jp/en/	General, iPS, and stem cell line
Institute for Frontier Medical Sciences^a^	shigen.nig.ac.jp/escell/human	Embryonic stem cell
Institute of Medical Science^a^	www.ims.u-tokyo.ac.jp/imsut/en/	Human cord blood stem cells
National Center Biobank Network	www.ncbiobank.org	Tissue, iPS cell, serum, plasma, PBMC, DNA
Tohoku Medical Megabank Organization (ToMMo) biobank	megabank.tohoku.ac.jp/english/	Serum, plasma, buffy coat, PBMC, urine, saliva, breast milk, DNA

^a^Biobanks funded by the third phase of the National BioResource Project (NBRP). Some institutes, such as RIKEN BRC, have assumed numerous NBRP projects but they are listed separately as each project has a clear organizational structure and hosts its own website

### China

Human biobanking in China launched in 1994 with the establishment of immortalized cell lines from Chinese ethnic groups (Gan et al. 2015). As the Chinese government and academia saw growing importance of biological resources, biobanking activities in China have significantly expanded in the last decade. Disease-oriented biobanks are often affiliated with hospitals in major cities, like Beijing and Shanghai, and equipped with advanced medical equipment (Zhang et al. 2015). Beijing Biobank of Clinical Resources (BBCR) is composed of 14 hospitals and considered as the largest clinical biobank network in China. The project team of BBCR aims for diverse management, standardized operation, and third-party supervision (Wang et al. 2015). With accumulated expertise on biobanking operation, BBCR also provides frameworks for constructing new biobanks. Shanghai also has a well-established biobank project, called Shanghai Biobank Network (SBN). Currently, SBN consists of 18 allied institutes (Zhang et al. 2015) and preserves liver cancer tissue and rheumatism samples as key collections among other diverse specimens (Fan and Zhang 2011). Population-based biobanks in China collect specimens from various groups of population based on the unique objective of each biobank. The China Kadoorie Biobank (CKB) began in 2003 to collect medical data and blood samples from 510,000 individuals in 10 geographic regions for research on common chronic diseases among Chinese people. Other prominent population-based projects are the Guangzhou Biobank Cohort Study on elderly people and the Born in Guangzhou Cohort Study which will follow 1,000,000 pregnant women and their offspring for 20 years (Zhang et al. 2015).

Like the preceding two countries, genetic resources of livestock and crops are actively managed by the Ministry of Agriculture (MOA). Chinese Academy of Agricultural Sciences (CAAS) is a major institute organizing and conducting agricultural research under the supervision of MOA. The Chinese central government has guaranteed financial support of CAAS under the Agricultural Science and Technology Innovation Program (ASTIP; American Association for the Advancement of Science 2013). The dedicated funding helps the institute continues on long-term preservation of agricultural resources and conducts rigorous research. CAAS is running a long-term nationwide biobank of plant germplasms and 10 medium-term plant biobanks.

The Ministry of Environment Protection (MEP) is mainly focused on maintaining biological diversity and protecting endemic and endangered species of China. The ministry endeavors to protect diverse organisms through a number of institutes, including the Southwest China Germplasm Bank of Wild Species (The Ministry of Environmental Protection of China 2014). This bank preserves plant seeds of 10,000 species, animal germplasms, macrofungi, and microorganisms. In addition, Chinese academic institutes now explore the cryopreservation option for cell lines of wild animals, such as Bengal tigers (Guan et al. 2010) and pandas (Yu et al. 2015).

The Ministry of Science and Technology (MOST) has established the National Science and Technology Infrastructure (NSTI) to raise research capacity by effective management of research resources (Ministry of Science and Technology of the People´s Republic of China 2006). NSTI aims to create a science and technology “infrastructure platform” which consolidates management system for effective use and sharing of biological resources and for prevention of wasted funding due to duplicated or unnecessary financial support. Under this project, an open database of biological resources in China is under development for improved sharing of resources and data (Xu 2007). One of the NSTI divisions is the National Infrastructure of Microbial Resources (NIMR) which collectively manages nine microbial resource centers. Each biobank has different characteristics and research emphasis as shown in their collections, either subject-specific (e.g. agricultural, medical, or pharmaceutical) or site-specific (e.g. marine-oriented). China General Microbiological Culture Collection Center (CGMCC) is one of the NSTI-supported biobanks geared toward more general and comprehensive collections of microorganisms and managed by the Institute of Microbiology at Chinese Academy of Science (IMCAS).

In order to create an integrated and open platform for effective management of biological resources, the Chinese government created China National Genebank (CNGB). The Genebank consists of bioresource bank, bioinformatics database, and consortium system (Zhang et al. 2015). Bioinformatics data are stored in cloud for various applications, such as healthcare and germplasm preservation (China National Genebank 2015). CNGB also engages in developing and providing standard operation procedures and training personnel for biobanking skills. The Genebank aspire to present a new biobanking model for the Chinese science communities and industries. Table 3 summarizes Chinese biobanks funded by the government.

**Table 3:** Biobanks in China

Name of institute	Website	Resources
China Center for Type Culture Collection	www.cctcc.org	Fungi, bacteria, algae, plant and animal cell line, virus
China Germplasm Bank of Wild Species	www.genobank.org	Fungi, seed, plant and animal germplasm, microorganism, DNA
China National GeneBank (CNGB)	www.nationalgenebank.org/en/	Animal, plant, microorganism, human-origin, metagenome data
*Animal and plant*		
Chinese Academy of Agricultural Sciences (CAAS)	www.caas.cn	Livestock and crop germplasm
Institute of Botany, Chinese Academy of Sciences	english.ib.cas.cn	Plant specimen, seed, fossil sample
Institute of Hydrobiology, Chinese Academy of Sciences	english.ihb.cas.cn	Aquatic organism, algae culture
China Zebrafish Resource Center	zfish.cn	Zebrafish
National Infrastructure of Fishery Germplasm Resources	zzzy.fishinfo.cn	Specimen, cell, sperm, DNA
*Microorganism*		
Agricultural Culture Collection of China	www.accc.org.cn	Fungi, plant pathogen
China Center for Industrial Culture Collection	www.china-cicc.org	Fungi, bacteria
China Forestry Culture Collection Center	www.cfcc-caf.org.cn	Fungi, bacteria, virus
China General Microbiological Culture Collection Center	www.cgmcc.net	Bacteria
China Pharmaceutical Culture Collection	www.cpcc.ac.cn	Fungi, bacteria, virus
China Veterinary Culture Collection Center	www.cvcc.org.cn	Bacteria
Institute of Microbiology, Chinese Academy of Sciences (IMCAS)	www.im.cas.cn	Fungi, virus, strain
Marine Culture Collection of China	www.mccc.org.cn	Fungi, bacteria
National Center for Medical Culture Collections	www.cmccb.org.cn	Bacteria
Wuhan Institute Of Virology, Chinese academy of Sciences	english.whiov.cas.cn	General and pathogenic virus
*Human-origin* ^a^		
Beijing Biobank of Clinical Resource	www.beijingbiobank.cn	Cell line, DNA
China Kadoorie Biobank	www.ckbiobank.org	Blood
China Marrow Donor Program	www.cmdp.com.cn	Blood
Clinical Oncology Institute, Beijing Cancer Hospital	www.bjcancer.org	Tumor tissue, blood
Eastern Hepatobiliary Surgery Hospital, the Second Military Medical University of Chinese PLA	www.ehbh.cn	Tissue, blood, urine, nucleic acid
Eighth Hospital of Wuhan City	www.wh8yy.cn	Tissue, blood, plasma, serum, fluid, nucleic acid, protein
Fudan-Taizhou Institute of Health Sciences	–	DNA
Guangxi Zhuang Autonomous Region Tumour Hopstial	www.gxhospital.com	Tissue, serum, plasma, lymphocyte
Jiangsu Province Hospital	www.jsph.net	Tumor tissue, blood
Renji Hospital, Shanghai Jiao Tong University	www.renji.com	Serum, plasma, tissue and DNA
Sixth Affiliated Hospital of Sun Yat-sen University	www.zs6y.com	Tissue, serum, plasma, blood cells, blood, feces
Sun Yat-sen University Cancer Center	www.sysucc.org.cn	Blood, serum, plasma, cell, nucleic acid
Taizhou Hospital of Zhejiang Province	www.tzhospital.com	Tissue, blood, serum, plasma, cerebrospinal fluid
The Fifth People’s Hospital of Shanghai	www.5thhospital.com	Blood, urine
The First Affiliated Hospital of Xinjiang Medical University	www.xydyfy.cn	Tumor tissue, blood
The Key Laboratory of Xinjiang Endemic, Ethnic Disease	–	Blood
Tianjin Medical University Cancer Institute & Hospital	www.tjmuch.com	Tumor tissue, blood
Tissue Bank of Fudan University Shanghai Cancer Center	www.shca.org.cn	Tissue
Tongji Hospital	www.tjh.com.cn	Tissue, blood, cell, DNA
West China Hospital	www.cd120.com	Tissue, blood
Wuxi No. 4 People’s Hospital (Affiliated Hospital of Jiangnan University)	www.wuxihospital.com	Tissue, RNA, serum, plasma, lymphocyte, stem cell and cell line
Zhejiang Cancer Hospital	www.zchospital.com	Tissue, tumor, serum, plasma, white blood cell layer

^a^The list of human-origin biobanks is adopted from Cheng et al. (2013)

## Other Asian biobanks and Asian biobank networks

Biobanks of other Asian countries are also in active operations. Most Asian countries have nationally funded culture collections available to international scholars. Some countries, such as Taiwan and Malaysia have population-based biobanking projects established to understand common and chronic diseases of their citizens. Table 4 is the partial list of Asian biobanks in Asia–Pacific.

**Table 4:** Biobanks in Asia–Pacific

Country	Name of institute	Website	Resources
*Animal and plant*			
India	National Bureau of Plant Genetic Resources	www.nbpgr.ernet.in	Plant genetic Resource
Taiwan	National Museum of Marine Biology and Aquarium	www.nmmba.gov.tw/english	Animal and algae specimen
	National Plant Genetic Resources Center	www.npgrc.tari.gov.tw	Crop germplasm
	World Vegetable Center	avrdc.org	Seed
Thailand	Siriraj House Dust Mite Center for Services and Research	www.dustmitethailand.com	Dust mite
Vietnam	Institute of Ecology and Biological Resources	www.iebr.ac.vn/index.asp?prgID=100	Animal and plant specimen
*Microorganism*			
Indonesia	Indonesian Center for Biodiversity and Biotechnology	icbb.or.id	Microorganism for plant
Malaysia	Malaysian Agriculture Research and Development Institute	www.mardi.gov.my	Bacteria, fungi, plant seed
Philippines	Microbiological Research and Services Laboratory Culture Collection, University of the Philippines-Diliman (UPCC)	nsri.upd.edu.ph/main/?page_id=140	Fungi and bacteria
Taiwan	Bioresource Collection and Research Center	www.bcrc.firdi.org.tw	Fungi and bacteria
Thailand	*Thailand Network on Culture Collection*	www1a.biotec.or.th/TNCC	
	Thailand Bioresource Research Center (TBRC)	www.tbrcnetwork.org	Fungi
	Department of Medical Sciences Thailand Culture Collection	engweb.dmsc.moph.go.th	Human pathogenic microorganism
	Plant Pathology and Microbiology Division, Department of Agriculture	www.doa.go.th/en/	Plant pathogenic microorganism
	Thailand Institute of Scientific and Technological Research	www.tistr.or.th/tistreng/	Industrial microorganism
Vietnam	Vietnam Type Culture Collection	vtcc.imbt.vnu.edu.vn	Fungi and bacteria
*Human*-*origin*			
Australia	Australasian Biospecimen Network Association	www.abna.org.au	Tumor
	Australian Breast Cancer Tissue Bank	www.abctb.org.au	Breast cancer tissue
India	ICMR National Tumor Tissue Repository, Tata Memorial Centre	tmc.gov.in	Tumor bone, blood, tissue
	Rajiv Gandhi Cancer Institute & Research Centre (RGCI & RC) Biorepository	www.rgcirc.org	Blood
Malaysia	The Malaysian Cohort	mycohort.gov.my	Blood, urine
Singapore	NUH Tissue Repository	medicine.nus.edu.sg/tissue/	Blood, body fluid, tissue
	Clinical Trials Resource Centre, Singapore General Hospital	www.sgh.com.sg	Tissue
	SingHealth Tissue Repository	research.singhealth.com.sg	Tissue, serum
Taiwan	Taiwan Biobank	www.twbiobank.org.tw	Blood, urine, DNA, plasma
Thailand	Tissue Repository of Chulabhorn Hospital	www.cccthai.org/l-eng/	Tissue

As numerous definitions of biobanks point out resource sharing as one of their key functions (Organisation for Economic Co-operation Development 2012; Hewitt and Watson 2013), networking is seemingly an indispensable element for most of the biobanks. Biobanks can improve their institutes and others by sharing their resources, data, and expertise through domestic and international networks. Generally, domestic biobanking networks in Asia are created or supported by governmental agencies for integrated management of specific resources, efficient funding, and support for research based on these resources. International networks are often more comprehensive in terms of resource types and aim for research cooperation at national-level. As domestic networks were described in previous section, we will focus on international networks in this section (Table 5).

**Table 5:** Biobank networks in Asia

Network name	Acronym	Homepage
Asian Network of Research Resource Centers	ANRRC	www.anrrc.org
ASEAN Centre for Biodiversity	ACB	www.aseanbiodiversity.org
Asian Consortium for the Conservation and Sustainable Use of Microbial Resources	ACM	www.acm-mrc.asia
Asian Biological Resource Center Network	ABRCN	www.abrcn.net

Asian Network of Research Resource Centers (ANRRC) is a well-established network of Asian and Oceanian biological and non-biological resource centers. In 2009, the first meeting of ANRRC was held in Korea. Since then, the network grew to include 103 centers from 14 countries (Lee et al. 2016). Through the network, members share biobanking expertise and scientific technology and open the prospect for collaboration. The 2015 annual meeting had a session dedicated to the standardization of biobanks to generate discussions and draw a consensus among Asian biobanking stakeholders.

For cooperation of Asian microbiological biobanks, Asian Consortium for the Conservation and Sustainable Use of Microbial Resources (ACM) is established in 2004. ACM has members from 13 Asian countries, including China, India, Indonesia, Japan, and Korea (Asian Consortium For the Conservation and Sustainable Use of Microbial Resources 2015b). In order to bolster international research collaboration, ACM encourages the establishment of proper microbial resource centers and provides training for preservation techniques and taxonomy of microorganisms (Asian Consortium For the Conservation and Sustainable Use of Microbial Resources 2010; Korean Collection for Type Cultures 2015). In addition, Asian Biological Resource Center Network (ABRCN) was organized for the establishment of database for sharing resources among ACM members (Asian Biological Resource Center Network 2015).

## Conclusion

Biobanking is the fundamental scientific effort to preserve and manage biological resources rapidly dissipating from the planet. In this paper, we have observed public biobanks of Asian countries, especially ones in China, Japan, and Korea. These biobanks are characterized by strategic operations orchestrated and assessed by their governments. Asian biobanks are also active in creating and utilizing networks for domestic and international collaboration. Recently, these three countries have enthusiastically partaken in the establishment of the International Standard for biobanks by TC 276 Biotechnology of the International Organization for Standardization (ISO).

In general, East Asian biobanks are heavily influenced by visions and plans of the governments as their financial resource is often solely dependent on governmental funding. Such fiscal system allows stable funding, however only for the defined period of time. Biobanks of Asia must consider diversifying funding sources by adding values to biological resources and offering additional services, such as data analyses, trainings, and software development as suggested by the OECD guideline for biological resource centers. Biobanks of Korea, Japan, and China have seen rapid growth in size over the last decade thanks to the substantial investment from governments. Now these countries should further focus on establishing stable and systematic resource centers and providing end-users with high-quality resources processed in compliance to international standards.

## Footnotes

1 Currently the Ministry of Science, ICT, and Future Planning (MSIP).
